# Building new approaches to care in Assisted Reproduction: how can we go farther?

**DOI:** 10.5935/1518-0557.20170015

**Published:** 2017

**Authors:** Maria do Carmo Borges de Souza, Roberto de Azevedo Antunes, Marcelo Marinho de Souza, Marcia Christina Gonçalves Gusmão

**Affiliations:** 1 Fertipraxis RJ, Human Reproduction Centre, Rio de Janeiro - RJ, Brazil

Assisted Reproduction specialists are quite aware of the issues derived from infertility. However, it is our point of view and understanding that failure to provide good patient care significantly affects our ability to develop sound relationships with them. Most Latin American countries follow the principles concerning reproductive health dictated by the WHO (^UN Women, 1995^), which provide for a comprehensive understanding of the matter and reinforce the right women have to a state of complete physical, mental, and social wellbeing as a vital condition for them to play an active role in every area of their public and private lives.

Inequality is a frequent issue affecting men and women in Latin America. Nonetheless, women occupy an even weaker position inside our family settings. Women in Latin America are often precluded from having access to health and wellbeing, for myriad reasons linked to geographic, social, political, economic, and biologic factors (^Souza & López, 2015^). Although there has been some progress in this area, our patients are still a long way from representing themselves and making decisions on their own, even in the context of assisted reproduction. The implementation of a new agenda requires support from all levels.

In daily practice, patients are insufficiently involved in choosing between treatment options (^van Empel *et al.*, 2010^), as treatment decisions are predominantly made by professionals based on their own diagnosis of a patient's physical condition. It has been proposed that not only the patient's physical condition, but also the patient's treatment preferences should be taken into account when choosing from different treatment options (^Mulley *et al.*, 2012^).

The notion of empowering the patient came from the ideas of Brazilian educator Freire (^Gadotti *et al.*, 1995^): "To me, critical reading basically implies that the reader is an intelligent individual, capable of decrypting the text. In this sense, the critical reader is he who 'rewrites' what he reads, who 're-creates' himself on his own terms. A non-critical reader functions as a kind of instrument of the author, a patient and docile repeater of what he reads." And the practical implications of this notion prescribe that one should not pre-define or restrict the roster of disease-or treatment-related outcomes, but rather discuss and negotiate with every patient or couple based on their particular situations and priorities in life.

Best practice may have different meanings when analyzed from the standpoint of experts or patients. According to ^Dancet *et al.* (2013)^, safety and effectiveness are the main goals of treatments to physicians, embryologists and nurses, while patients care more about being in the center of care, which means they desire attention during the course of treatment.

But how should we look at this? There certainly is some tension in the air, as we do not have the right answer to every patient nor a definitive protocol suitable for everyone.

The roster of procedures available today includes individualized controlled ovarian stimulation (ICOS), fresh and frozen embryo transfers, preimplantation genetic screening (PGS), and fertility preservation, to name a few. But which of these are possible or acceptable options for our patients? We cannot assume that the choice we would make is necessarily the choice our patients would make.

Many women have elected to have their oocytes frozen to increase their chances of getting pregnant after their fertility has declined. Financially independent women living alone in urban areas have opted for oocyte cryopreservation. However, this does not mean that they are ready to become single parents should prince charming not knock on their doors. According to ^de Groot *et al.* (2016)^, patients given information about the procedure's limited success rates and potential risks, perceived oocyte cryopreservation as a "helping hand" to achieving shared parenthood. They were willing to spend money with fertility treatment because the prospect of shared parenthood outshone the perceived health risks and burden.

Being open to and accepting change is easier said than done, and involves an effort that requires constant training and reassessment of our multidisciplinary teams. As part of our quality management efforts, ART centers should try to offer truly patient-centered care, in addition to the customary measures to enhance procedure safety and effectiveness. The time has come for us to listen to our patients before decisions are made. Our staff's creativity and our patients' ideas must be discussed as potential paths to improved quality and novel care practices. And as Paulo Freire once wrote, "It is of the essence to bridge the gap between what is said and what is done, so that at any given time your discourse mirrors your practice."
